# Nurses' Scope of Practice in Home‐Based Care: An Observational Study of Care Activities, Clinical Decision‐Making, and Contextual Modulators—The DECIDE Project

**DOI:** 10.1111/jocn.70451

**Published:** 2026-07-22

**Authors:** Ida Røed Flyum, Gunilla Borglin, Anna Josse Eklund, Veronica Pavedahl, Edith Roth Gjevjon

**Affiliations:** ^1^ Department of Undergraduate Studies Lovisenberg Diaconal University College Oslo Norway; ^2^ Department of Nursing, Faculty of Health, Nature and Technical Science, Institute of Health Sciences Karlstad University Karlstad Sweden

**Keywords:** clinical decision‐making, contextual factors, healthcare workers, home care services, professional autonomy, registered nurses, scope of practice

## Abstract

**Background and Aim:**

Nurses' scope of practice is central to healthcare delivery. While scope of practice is well described conceptually, there is limited empirical research capturing how it is enacted in everyday clinical practice. This study aimed to describe scope of practice in home‐based care, focusing on care activities, clinical decision‐making, professional autonomy and contextual modulators.

**Design:**

A multi‐method observational design.

**Setting:**

Three nursing teams within home healthcare services in a Norwegian municipality—the DECIDE project.

**Methods:**

Quantitative and qualitative data were collected through structured observations guided by a conceptual framework. Data were analysed using descriptive statistics and qualitative categorisation.

**Results:**

A total of 827 care activities were observed across 239 observations. Care activities most often addressed physical and medical needs, while psychosocial needs were less frequently observed. Registered nurses more often engaged in both direct and indirect care and primarily delivered compensatory care. Clinical decision‐making was most frequently observed during assessment, while other phases of the nursing process, particularly evaluation, were less evident. These patterns were consistently associated with contextual modulators observed during care delivery.

**Conclusions:**

Nurses' scope of practice in home‐based care is enacted through care activities and emerges as a context‐sensitive, system‐level phenomenon shaped by contextual modulators. Registered nurses frequently performed tasks beyond planned visits, while non‐registered nurses concentrated on activities of daily living. Both groups showed limited engagement with the evaluation phase of the nursing process.

**Implications for the Profession and/or Patient Care:**

Strengthening clinical leadership, decision‐making autonomy, and organisational support may support enactment of scope of practice and improve care quality in home‐based settings.

**Reporting Method:**

The study followed reporting standards for mixed‐methods research and was appraised using the Mixed Methods Appraisal Tool.

**Patient or Public Contribution:**

Patient and public involvement was incorporated at the DECIDE project level but not in this observational study.

## What Does This Paper Contribute to the Wider Global Clinical Community?

- Provides empirical insight into how nurses' scope of practice is enacted in real‐world clinical settings, rather than inferred from policy or role descriptions
- Demonstrates how contextual modulators, including time constraints, interruptions, and organisational conditions, shape nursing practice across care settings
- Identifies a consistent gap in the evaluation phase of the nursing process, with implications for care quality and patient safety internationally
- Supports understanding of scope of practice as a context‐sensitive, system‐level phenomenon
- Offers an observational approach to studying clinical practice that may inform future research and practice development in diverse care contexts

## 1 Introduction

Home‐based care relies heavily on nurses to deliver and coordinate care for older people with complex needs. To provide tailored and competent services, nurses must collaborate effectively while maintaining clear professional boundaries within the multifaceted home‐based care context (Higgs et al. 2008). In this study, Registered Nurses are referred to as RNs, whereas healthcare workers with vocational training but without RN licensure are referred to as non‐RNs. Within the Norwegian home‐based care context, these personnel constitute an integral part of the nursing workforce. When both groups are discussed together, the term nurses is used. (see Table S1 for this study's core constructs). The scope of practice (SoP) for RNs has been described as encompassing their responsibilities and authority (role), the activities and tasks they perform (function), and their capacity for clinical decision‐making (Malone and Cliffe 2018; Royal College of Nursing [RCN] 2023), including responsibilities related to coordination, delegation, and clinical leadership (cf. Begley and Hughes 2023). How this conceptualisation applies to non‐RNs, and how RNs' SoP is enacted in relation to non‐RNs under the contextual constraints that can characterise home‐based care, remains underexplored. Both RNs and non‐RNs were included, as nursing practice in home‐based care is inherently interdependent and context‐bound. Within this study, SoP is conceptualised through nurses' roles, functions, and clinical decision‐making. These components are enacted through observable care activities, application of the nursing process, performance level of care, and professional autonomy. The enactment of SoP is further shaped by contextual modulators, understood as factors influencing practice at individual, team, and organisational levels. Examples include leadership, organisational structures, and nursing unit culture. Together, these core constructs provide a conceptual framework for examining how nurses' SoP can be described in home‐based care. The relationship between these constructs is illustrated in Figure 1 and further operationalised in Table S1.

**FIGURE 1:**
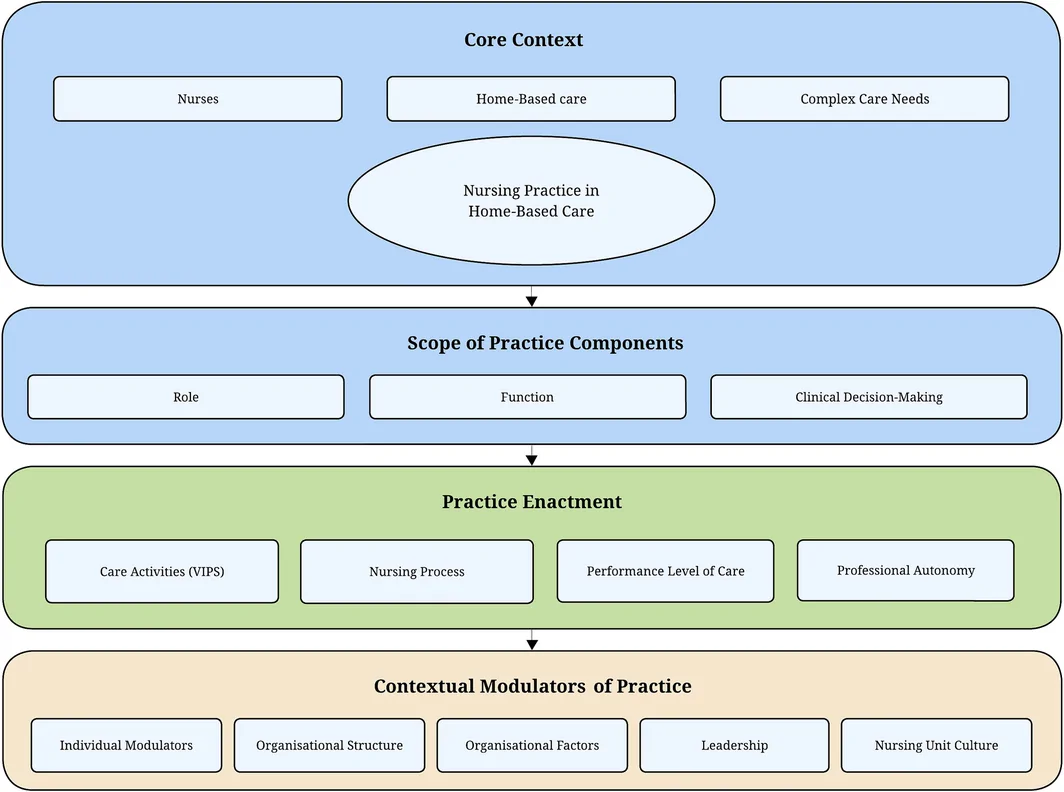
Operationalisation of core constructs and analytical structure. [Colour figure can be viewed at wileyonlinelibrary.com]

Given the characteristics of the older population receiving home‐based care, care activities commonly address physical, psychosocial, and medical needs (Ehnfors et al. 2015). Yet meeting these needs is rarely straightforward. RNs must balance direct clinical care with responsibilities for coordination and clinical leadership across nursing services (RCN 2023). This dual role demands high levels of competence and positions RNs as the linchpin of holistic and tailored home‐based nursing care. Alongside RNs, non‐RNs constitute a substantial proportion of the home‐based care workforce (Flodgren and Meneses 2017). They are often described as valuable extensions of RNs (cf. Richards and Borglin 2019), primarily responsible for providing and monitoring direct care while reporting to RNs, who retain responsibility for clinical leadership and decision‐making (Nordaunet et al. 2025; RCN 2023). A key component of the RN's autonomous role (responsibility) is clinical decision‐making, which is commonly operationalised through the nursing process, encompassing assessment, diagnosis, planning, implementation, and evaluation of care (Ranta and Kaunonen 2024; Thompson and Stapley 2011). This involves determining what care is needed, when to act, and how to proceed. Because clinical decision‐making is not directly observable, the nursing process provided us with an established framework through which decision‐making could be identified and examined in practice. Clinical decision‐making becomes especially critical in home‐based settings, where nurses often work independently and face time constraints, decentralised service delivery, and unpredictable care environments (Brenne et al. 2022). These conditions highlight the complexity of nursing practice and the need for a realistic understanding of nurses' SoP. Such understanding is important for informing relevant clinical developments in home‐based care.

Henderson's seminal description of nursing (1997) has profoundly shaped how the role and function of RNs are operationalised today. Her work introduced foundational concepts such as professional autonomy and performance level, which have since been expanded by scholars like Wilkinson (2011). Here, performance level of care refers to the degree of support provided by the nurse, ranging from compensatory care to collaborative care aimed at maintaining patient independence. According to Henderson, autonomy involves tailoring care to meet the individual needs of recipients while preserving their independence by offering only the support that is necessary. While her focus was primarily on direct care, Wilkinson extended the concept of autonomy to include broader dimensions of SoP, emphasising how contextual modulators can either support or constrain nurses' autonomy.

One such modulator is nurses' clinical leadership, which is closely linked to RNs' autonomous SoP and is recognised as a core responsibility of the profession (Cope and Murray 2017; Ranta and Kaunonen 2024). Clinical leadership distinguishes RNs from non‐RNs, not only through their formal authority to delegate care, but also through their responsibility to coordinate care, ensure quality, and share knowledge within the team. Importantly, this clinical leadership is not confined to those in formal managerial roles. Drawing on contemporary perspectives, we argue that every RN, regardless of title, must act as a clinical leader in their practice (Cline et al. 2022). Recent studies suggest that a lack of clear clinical leadership and mutual understanding between RNs and non‐RNs can lead to fragmented care and diminished quality (Nordaunet et al. 2025; Sandberg et al. 2026).

Despite its foundational importance, nurses' SoP is increasingly shaped by contextual modulators. The latter include individual factors such as clinical competence, self‐awareness, stress levels, and leadership style (Cope and Murray 2017; Lister et al. 2020), and organisational elements like service structures (Orvik 2022) and cultural norms (Callen et al. 2009). Emerging research suggests that examining nurses' SoP in isolation is insufficient (Nordaunet et al. 2025; Sandberg et al. 2026). Rather, SoP appears highly sensitive to its context, aligning with Skivington et al.'s (2021) conceptualisation of it as a system event—that is, a phenomenon shaped by interactions between individuals, teams, and organisational conditions rather than through isolated actions or decisions. This perspective highlights the importance of research that accounts for both systemic and situational modulators when examining practice. Given the nuanced and multifaceted nature of nurses' SoP in home‐based care, it is essential for research to engage directly with clinical environments. Observational methods are particularly well suited to capturing real‐time clinical decision‐making, everyday care practices, and contextual modulators that may not be fully revealed through self‐report or interview‐based approaches. Therefore, this study aimed to describe nurses' scope of practice in home‐based care, focusing on care activities, clinical decision‐making, professional autonomy, and contextual modulators of practice.

## 2 Methods

### 2.1 Study Design

A multi‐method observational design was employed. The study was guided by a conceptual framework outlining SoP as comprising role, function, and clinical decision‐making, enacted in practice through care activities, the nursing process, performance level of care, and professional autonomy, and shaped by contextual modulators (Figure 1). Quantitative and qualitative data were collected through direct structured observations (Fix et al. 2022), including field notes (Patton 2015). Numeric data were analysed using descriptive and comparative statistics (Field 2024). Textual data, namely, the field notes, were thematically sorted and used to illustrate patterns in the numerical findings (Nordaunet et al. 2025). This study was reported in accordance with relevant guidelines for mixed‐methods research and appraised using the Mixed Methods Appraisal Tool (MMAT: Table S2) (Hong et al. 2018).

### 2.2 Research Context and Settings

This study was conducted within three home‐based nursing teams in a larger Norwegian municipal home healthcare service. Home‐based care refers to health and care services delivered in individuals' homes and encompasses a broad range of preventive, curative, rehabilitative, supportive, and palliative services provided across the life course. Although the concept is widely used, its definition and organisation vary across healthcare systems and national contexts. In Norway, however, home‐based care services are predominantly directed toward older people with chronic illness, multimorbidity, and functional limitations who require support to remain living at home.

Care is mainly delivered by nurses. RNs complete a three‐year bachelor's degree in general nursing science, which confers both academic credentials and professional licensure required for RN practice in Norway. Non‐RNs broadly correspond to healthcare assistants, nursing assistants, or similar support‐care roles in other healthcare systems, although professional titles, educational pathways, and role and function vary internationally. In Norway non‐RNs undergo 2 years of vocational training at the upper secondary level, followed by an apprenticeship, leading to a trade certificate and licensure. By law, each municipality is responsible for organising and funding home‐based nursing services. A local administrative unit, separate from care providers, assesses the needs of individuals applying for services and determines which care activities can be granted based on this assessment. As a result, care activities delivered by nurses are strictly regulated. Each visit is pre‐planned with specified activities with a set timeframe. Where the latter is based on the number and complexity of the care activities.

### 2.3 Sampling and Recruitment Strategies

A sequential multistage sampling technique was employed for recruitment. First, one home healthcare service comprising six nursing teams in a large municipality in South‐Eastern Norway was recruited. Second, three independent nursing teams were randomly selected using a simple random sampling procedure. Although the nursing teams belonged to the same municipal home healthcare service, they operated as independent units. Each team was responsible for organising and delivering care within its designated geographical area. This enabled variation in team composition, workflow, leadership practices and local care organisation, while maintaining an overarching organisational context. Our sampling strategy allowed examination of practice variation at team level without introducing unnecessary differences in overarching policy or service structure. Oral and written information was provided to the team leaders (RNs) and the service manager. Third, nurses and care recipients affiliated with the selected teams were recruited through purposive sampling (Patton 2015). Participation was voluntary, and recruitment was facilitated through team leaders and professional development RNs. All consent procedures were handled individually by the first author (IRF) to minimise potential coercion. Informational meetings were held and flyers were posted in the teams' break rooms, including contact information and QR codes. Each observation period was preceded by two days of familiarisation with the teams.

Inclusion criteria for nurses were permanent or long‐term temporary employment, direct involvement in nursing care for older people with complex needs, and proficiency in Norwegian. Including both RNs and non‐RNs enabled examination of RNs' SoP as it is enacted within the broader care context. As nursing work in home‐based care involves coordination, delegation, and collaboration across staff categories, observing only RNs would not have captured the conditions shaping their practice. Recruitment therefore prioritised RNs (approximately 80%), while allowing inclusion of non‐RNs to support contextual understanding. The final distribution (approximately 75% RN and 25% non‐RN observations) was considered appropriate and consistent with the study aim. Care recipients were eligible if they were aged 65 or older, presented with complex care needs (i.e., ≥ 2 co‐occurring diagnoses as assessed by the RNs), received daily home‐based nursing care, and were proficient in Norwegian. The RNs assessed care recipients' capacity to provide informed consent. Where this was not possible, proxy consent was obtained from a close relative following a benefit–risk assessment. Care recipients received written and oral information about the study prior to observations, and informed consent was obtained before participation (Table 1).

**TABLE 1:** Study setting and data collection context.

	Home healthcare service area^a^		
	Team A	Team B	Team C
Older people receiving home‐based care per team, *n*	99	124	94
Physical presence on site per team (hours)	≈75	≈72	≈71
Direct structured observations per team (hours)	≈27	≈26	≈27
Nursing staff^b^ per observed team, *n*			
RNs	14	9	8
Non‐RNs	11	9	7

Abbreviations: *n* = a sample of the total number, *N* = total number, non‐RN = non‐Registered Nurse, RN = Registered Nurse.

^a^ Older people (*N* = 617) receiving home‐based nursing care within the service area (*N* = 6 nursing teams in total).

^b^ RNs (*N* = 56) and non‐RNs (*N* = 58) within the service area.

### 2.4 Data Collection

Structured direct observations (Fix et al. 2022) were conducted by the first author (IRF), an RN. The observer adopted a non‐participant role; hence, interactions with participants were limited. Structuring the observations ensured consistency in what was and was not documented while allowing unexpected findings to be captured in field notes (Fix et al. 2022). Observations were conducted over 10 to 12 days per team between January and June 2025. Data were gathered during day and evening shifts (08:00–15:00 and 15:00–22:30) on weekdays and weekends, with the observer present for the entire duration of each shift. Observations commenced when the nurses exhibited a cue (a signal, hint, or prompt) in the protocol to engage in a care activity across the VIPS model's (Well‐being, Integrity, Prevention and Safety) physical, psychosocial, or medical domain (Ehnfors et al. 2015) and ended when the activity was completed.

The protocol consisted of four sections comprising observational cues for the study framework (Figure 1). The first section (A–B) included the VIPS model, which served as the main unit of observation (Ehnfors et al. 2015). The second section (C–D) captured observational cues related to the nurse's clinical decision‐making, as aligned with the nursing process (Wilkinson 2011). As several phases of the nursing process are cognitive, these were operationalised through observable cues, such as verbalisation, interaction and action. Field notes were used to capture implicit aspects of decision‐making where possible. The third section (E–F) included the performance level of the nursing activity (Henderson 1997) and the nurse's level of professional autonomy (Wilkinson 2011). The fourth and final section (G–I) captured contextual modulators, conceptualised as multi‐level influences on practice, including dynamic conditions (e.g., individual modulators and organisational factors; Lister et al. 2020) and stable organisational characteristics (e.g., organisational structure, leadership style and nursing unit culture; Cope and Murray 2017, Callen et al. 2009, Orvik 2022). Details regarding the observational protocol are provided in Table S3.

The observational protocol was informed by previously applied structured observation (e.g., Nordaunet et al. 2025; Sandberg et al. 2026), using comparable protocol structures. While these studies applied the Fundamentals of Care framework, the present study utilised the VIPS model to operationalise care activities across physical, psychosocial, and medical domains. The protocol structure has therefore been applied in both home‐based and facility‐based care settings without reported methodological concerns, supporting its relevance across care contexts. Prior to data collection, the protocol was field‐tested, resulting in no amendments.

### 2.5 Data Analysis

Numerical data were analysed using descriptive and comparative statistics (Field 2024) with IBM SPSS software (IBM Corp 2023). Descriptive statistics included frequencies, percentages, means and standard deviations. Comparative analyses were conducted between teams and between RNs and non‐RNs using chi‐square tests (χ^2^), with *p*‐values < 0.05 considered statistically significant (95% confidence interval). Only in‐text reporting is used for the chi‐square statistics. Textual data from field notes were thematically organised (Nordaunet et al. 2025) to contextualise and exemplify observed patterns. To enhance analytical rigour, coding and categorisation of observational data were regularly discussed within the research team. Ambiguities were reviewed collaboratively to ensure consistency and accuracy in data interpretation.

### 2.6 Ethical Considerations and Data Management

This study adhered to relevant ethical guidelines (World Medical Association 2024) and was conducted in accordance with the European Union's General Data Protection Regulation. Ethical approval was obtained from the Regional Committees for Medical and Health Research Ethics and the Norwegian Agency for Shared Services in Education and Research (IDs 7,33,476 and 3,49,256), as well as from the Research Ethics Committee at Karlstad University and the Swedish Ethical Review Authority (IDs C2024/636 and 2025‐00816‐01). The study was part of the DECIDE project, which includes patient and public involvement at programme level.

## 3 Results

The findings are presented from observed care activities and clinical decision‐making to professional autonomy and the contextual modulators shaping nursing practice.

### 3.1 Characteristics of the Observations

A total of 239 observations, corresponding to approximately 80 h, were conducted across the three nursing teams (Table 2), mainly during weekday morning shifts (08:00–15:00). Most observations (81%) lasted between 5 and 30 min (M = 20.15, SD = 13.4), with similar patterns across teams: Team A (78%, M = 21.32, SD = 13.30), Team B (87%, M = 18.7, SD = 10.87) and Team C (76%, M = 20.55, SD = 15.57). No significant differences were found between teams regarding duration.

**TABLE 2:** Characteristics of the observations.

Characteristics	Teams A–C	Team A	Team B	Team C
Observations, *N/n* (%)	239	75 (31.4)	84 (35.1)	80 (33.5)
Length of observations, *N/n* (%)				
≈1–15 min	106 (44.4)	28 (37.3)	37 (44.0)	41 (51.2)
≈16–30 min	87 (36.4)	31 (41.3)	36 (42.9)	20 (25.0)
≈31–45 min	31 (13.0)	11 (14.7)	9 (10.7)	11 (13.8)
≈46–60 min	15 (6.3)	5 (6.7)	2 (2.4)	8 (10.0)
Observations during weekdays and weekends, *N/n* (%)				
Monday – Friday	193 (80.8)	68 (90.7)	67 (79.8)	58 (72.5)
Weekends	46 (19.2)	7 (9.3)	17 (20.2)	22 (27.5)
Observations during morning and afternoon shifts, *N/n* (%)				
Morning shift	145 (60.7)	49 (65.3)	55 (65.5)	41 (51.2)
Afternoon shift	94 (39.3)	26 (34.7)	29 (34.5)	39 (48.8)
Care activities nursing staff engaged in, *N/n* (%)				
Care activities related to physical needs	573 (69.4)	149 (61.8)	227 (74.0)	197 (71.0)
Care activities related to psychosocial needs	83 (10.1)	37 (15.4)	21 (6.80)	25 (9.10)
Care activities related to composite‐ and medical assessments	171 (20.5)	55 (22.8)	59 (19.2)	57 (19.9)
Nurses part of the observations, *N/n* (%)				
RNs	182 (76.2)	56 (74.7)	59 (70.2)	67 (83.8)
Non‐RNs	57 (23.8)	19 (25.3)	25 (29.8)	13 (16.3)
Team composition in observations, *N/n* (%)				
Working alone	203 (84.9)	69 (92.0)	70 (83.3)	64 (80.0)
Working in pairs	31 (13.0)	6 (8.00)	11 (13.1)	14 (17.5)
Team > 3 nurses present	5 (2.1)	—	3 (3.6)	2 (2.5)
Observations of direct and indirect care activities, *N/n* (%)				
RN direct care activity	138 (57.7)	39 (52.0)	44 (52.3)	55 (68.8)
Non‐RN direct care activity	55 (23.0)	19 (25.3)	24 (28.6)	12 (15.0)
RN indirect care activity	44 (18.4)	17 (22.7)	15 (17.9)	12 (15.0)
Non‐RN indirect care activity	2 (0.80)	—	1 (1.20)	1 (1.30)
Observations of planned vs unplanned home visits, *N*/*n* (%)				
Planned visits	187 (78.2)	57 (76)	66 (78.6)	64 (80.0)
Unplanned visits	52 (21.8)	18 (24)	18 (21.4)	16 (20.0)
Participants, *N/n* (%)				
RN	20 (21.3)	7 (21.2)	7 (22.6)	6 (18.8)
Non‐RN	12 (12.8)	5 (15.2)	3 (9.7)	4 (12.5)
Older care recipients	64 (65.9)	21 (63.6)	21 (67.7)	22 (68.7)

Abbreviations: *n* = a sample of the total number, *N* = total number, Non‐RN = Non‐Registered Nurse, RN = Registered Nurse.

RNs accounted for 76.2% of observations, while non‐RNs accounted for 23.8%. RNs were observed engaging in care activities 76.2% of the time, most often working alone. Team A nurses worked alone in over 90% of observations, while teamwork was more common in Teams B and C. RNs were more frequently involved in both direct (care recipient present) and indirect (care recipient absent) care compared with non‐RNs (Table 2). No significant team differences were found in team composition or care type.

### 3.2 Observed Care Activities

Care activities, representing the functional component of practice enactment, were analysed using the VIPS model (Table 3). Across all observations, nurses engaged in 827 care activities (M = 3.46 per observation). In 32.6% of observations, care activities related to one VIPS area only; 50.2% involved two areas, and 17.2% involved all three. Team A more frequently engaged in all three VIPS areas (21.3%) compared with Teams B (15.5%) and C (15.2%).

**TABLE 3:** Observed care activities among nurses across teams.

Care activities (VIPS model)	Teams A–C	Team A	Team B	Team C
	(*N* = 239)	(*n* = 75)	(*n* = 84)	(*n* = 80)
Total number of observed care activities *N*/*n* (%)	827	241 (29)	307 (37)	279 (34)
Mean number of care activities per observation (SD)	3.5 (2.1)	3.2 (2.0)	3.7 (2.1)	3.5 (2.2)
Care activities related to physical needs, *N*/*n* (%)	573	149	227	197
Communication	16 (2.8)	1 (0.7)	6 (2.6)	9 (4.6)
Knowledge/development	39 (6.8)	11 (7.4)	19 (8.4)	9 (4.6)
Breathing/circulation	61 (10.3)	13 (8.7)	22 (9.7)	25 (12.2)
Nutrition	125 (21.9)	35 (23.5)	56 (24.7)	34 (17.3)
Elimination	91 (15.9)	27 (18.1)	29 (12.8)	35 (17.9)
Skin/tissue‐ wound	63 (11.0)	17 (11.4)	22 (9.7)	24 (12.2)
Activity (ADL/Mobility)	107 (18.7)	26 (17.4)	41 (18.0)	40 (20.4)
Sleep	33 (5.8)	10 (6.7)	17 (7.5)	6 (3.1)
Pain/perception	39 (6.8)	9 (6.1)	15 (6.6)	15 (7.7)
Sexuality/reproduction	—	—	—	—
Care activities related to psychosocial needs, *N*/*n* (%)	83	37 (44.6)	21 (25.3)	25 (30.1)
Emotions	36 (43.4)	17 (46.0)	8 (38.1)	11 (44.0)
Relations	28 (33.7)	13 (35.1)	7 (33.3)	8 (32.0)
Spiritual/cultural wellbeing	19 (22.9)	7 (18.9)	6 (28.6)	6 (24.0)
Care activities related to composite and medical assessments, *N*/*n* (%)	171	55 (32.2)	59 (34.5)	57 (33.3)
Composite assessment (objective assessments)	—	—	—	—
Medications	164 (95.9)	51 (92.7)	59 (100)	54 (96.4)
Medical assessment	7 (4.1)	4 (7.3)	—	3 (5.5)

Abbreviations: ADL = activities of daily living, *n* = a sample of the total number, *N* = total number, SD = Standard Deviation.

The most common care activities addressed physical needs (69.3%), followed by composite or medical assessments (20.7%) and psychosocial needs (10%). The proportion of physical care varied across teams (61.8% in Team A to 74% in Team B) (Table 3). Within physical needs, care activities focused primarily on nutrition, ADL/mobility, elimination, skin/wound care and breathing/circulation. Team C performed more ADL/mobility care and less nutrition‐related care than other teams. Nearly all composite/medical assessments (96%) concerned medication, and no composite assessments were recorded during the observations. Psychosocial care focused mainly on emotional needs, with limited representation of care activities addressing spiritual/cultural well‐being.

Statistically significant differences were found between teams for nutrition and communication: Team B had more nutrition‐related care activities (χ^2^[2, *N* = 239] = 10.984, *p* = 0.004) than Teams A and C, while Team A engaged less in communication. RNs were more likely than non‐RNs to engage in knowledge/development care activities and less likely to engage in care activities related to spiritual or cultural well‐being.

### 3.3 Clinical Decision‐Making

Clinical decision‐making, operationalised as the nursing process, was examined through observed cues across phases of care activities. During 182 RN‐only observations, 523 decision‐making cues were documented. Most related to physical needs (69%), followed by composite/medical (30%) and psychosocial (1%) needs. RNs typically engaged in three phases of the nursing process, most often ‘assessment’ (27.7%), followed by ‘implementation’, ‘planning/goal setting’, and ‘analysis/diagnosis’; ‘evaluation’ was least frequent (6.7%) (Table 4). RNs in Team C engaged less often in ‘analysis/diagnosis’ (χ^2^[2, *N* = 182] = 8.104, *p* = 0.017) than Teams A and B. Across all nurses, RNs were more frequently observed during the assessment and analysis/diagnosis phases than non‐RNs.

**TABLE 4:** Clinical decision‐making [process phases] among registered nurses.

Clinical decision‐making [process phases]	Teams A–C	Team A	Team B	Team C
	(*N =* 182)	(*n =* 56)	(*n =* 59)	(*n =* 67)
Total number of decision‐making cues, *N*/*n*	523	169	175	179
Mean number of decision‐making cues per observation (SD)	2.9 (1.1)	3.0 (1.1)	3.0 (1.1)	2.7 (1.1)
Decision‐making cues by process phase, *N*/*n* (%)				
Assessment	145 (27.7)	50 (29.6)	46 (26.3)	49 (27.4)
Analysis/diagnosis	101 (19.3)	35 (20.7)	38 (21.7)	28 (15.6)
Planning/goal setting	113 (21.6)	36 (21.3)	37 (21.1)	40 (22.3)
Implementation	129 (24.7)	35 (20.7)	42 (24.0)	52 (29.1)
Evaluation	35 (6.7)	13 (7.7)	12 (6.9)	10 (5.6)

Abbreviations: *n* = a sample of the total number, *N* = total number, SD = Standard Deviation.

Among RNs, Team C was less frequently observed in both the assessment and analysis/diagnosis phases compared with Teams A and B. Assessment was most associated with elimination care while analysis/diagnosis related to knowledge/development, elimination, skin/wound and sleep. Planning/goal setting was linked to activity/mobility and less to elimination. Evaluation, though rare, was more likely during knowledge/development, breathing/circulation, and elimination.

### 3.4 Professional Autonomy and Performance Level

Professional autonomy, reflecting decision‐making authority within care activities, was analysed in relation to observed practice. Professional autonomy and performance level of care varied across observations and teams, with interdependent care activities being most frequent overall, followed by independent and dependent activities (Table 5). Among RNs, professional autonomy varied, with 47.8% of care activities classified as interdependent (decided and informed in collaboration with other professionals), 33% as independent (decided and informed by the RNs themselves) and 19.2% as dependent (decided by other professionals). Team A RNs demonstrated greater independence and fewer interdependent care activities, while Team C RNs engaged more frequently in dependent activities.

**TABLE 5:** Professional autonomy and performance level of care among nurses.

	Teams A–C	Team A	Team B	Team C
	(*N* = 239)	(*n* = 75)	(*n* = 84)	(*n* = 80)
Professional autonomy, *N*/*n* (%)				
Independent activity	60 (33.0)	35 (62.5)	11 (18.6)	14 (20.9)
Interdependent activity	87 (47.8)	15 (26.8)	38 (64.4)	34 (50.7)
Dependent activity	35 (19.2)	6 (10.7)	10 (17.0)	19 (28.4)
Performance level of care, *N*/*n* (%)				
Compensating care	108 (45.2)	34 (45.3)	31 (36.9)	43 (53.8)
Partially compensating care	50 (20.9)	18 (24.0)	15 (17.9)	17 (21.3)
Collaborative care	81 (33.9)	23 (30.7)	38 (45.2)	20 (25.0)

Abbreviations: *n* = a sample of the total number, *N* = total number.

In terms of performance level (Table 5), nurses most frequently provided compensatory care (45.2%), followed by collaborative (33.9%) and partially compensatory care (20.9%). Compared with Teams A and C, Team B showed a higher proportion of collaborative care, although this difference was not statistically significant.

### 3.5 Contextual Modulators of Practice

Contextual modulators of practice were analysed as conditions influencing how care activities, decision‐making and autonomy were enacted in situ. These modulators encompassed both individual and organisational conditions observable during care delivery. Individual modulators reflected nurses' capability to perform, assess, concentrate, and make decisions, while organisational modulators captured conditions related to the organisation of care, including workload, interruptions and work environment. In addition, patterns related to organisational modulators such as structure, leadership and nursing unit culture were identified as shaping how practice was carried out in everyday care situations.

### 3.6 Individual Modulators

Across 182 RN‐only observations, 664 observable cues for individual contextual modulators were identified. RNs' practical and situational assessment skills were generally rated as ‘good’, with 8.8% rated ‘poor’ or ‘very poor’. Concentration and decision‐making abilities were also mostly ‘good’, though poor decision‐making was noted in 10.4% of cases (Table 6). Interruptions occurred in about 30% of observations, as illustrated in field notes describing RNs balancing urgent calls during care activities.

RNs' phone rings during personal hygiene activity. RN comments that the person on the other side will have to wait. Phone rings again, two times. The RN says to the older person and their colleague (RN, not participating in study) that it might be urgent and the RN will therefore have to answer. Goes to the hallway and calls back. It is an urgent matter, and RN therefore must confer for several minutes before re‐entering the room. The RN seems somewhat stressed but finishes the visit and keeps calm and focused on the older person. (Field note #203)

**TABLE 6:** Contextual modulators of practice among registered nurses.

Contextual modulators	Teams A–C			Team A			Team B			Team C		
	*N* = 182			*n* = 56			*n* = 59			*n* = 67		
Individual modulators (Poor/Acceptable/Good^a^), *N*/*n* (%)												
Capability to handle practical tasks	1 (0.5)	38 (20.9)	81 (44.5)	1 (1.8)	8 (14.3)	27 (48.2)	—	18 (30.5)	21 (35.6)	—	12 (17.9)	33 (49.3)
Capability to take note and assess the situation	16 (8.8)	75 (41.2)	91 (50.0)	4 (7.1)	19 (33.9)	33 (58.9)	6 (10.2)	23 (39.0)	30 (50.8)	6 (9.0)	33 (49.3)	28 (41.8)
Capability to concentrate during activities	18 (9.9)	42 (23.1)	122 (67.0)	3 (5.4)	13 (23.2)	40 (71.4)	6 (10.2)	12 (20.3)	41 (69.5)	9 (13.4)	17 (25.4)	41 (61.2)
Capability to make decisions in a situation	19 (10.4)	71 (39.0)	90 (59.5)	4 (7.1)	19 (33.9)	55 (57.1)	8 (13.6)	19 (32.2)	32 (54.2)	7 (10.4)	33 (49.3)	26 (38.8)
Organisational modulators (Never/Sometimes/A great deal^b^), *N*/*n* (%)												
Shows signs of/remarks stress	132 (72.5)	48 (26.4)	2 (1.1)	40 (71.4)	16 (28.6)	—	49 (83.1)	10 (16.9)	—	43 (64.2)	22 (32.8)	2 (3.0)
Shows signs of/remarks tiredness	159 (87.4)	22 (12.1)	1 (0.5)	52 (92.9)	4 (7.1)	—	52 (88.1)	7 (11.9)	—	55 (82.1)	11 (16.4)	1 (1.5)
Identifies and prevents risk	56 (30.8)	100 (54.9)	12 (6.6)	14 (25.0)	28 (50.0)	4 (7.1)	19 (32.2)	35 (59.3)	2 (3.4)	23 (34.3)	37 (55.2)	6 (9.0)
Information flow takes place between nurses	3 (1.6)	42 (23.1)	4 (2.2)	—	14 (25.0)	1 (1.8)	2 (3.4)	15 (25.4)	1 (1.7)	1 (1.5)	13 (19.4)	2 (3.0)
Co‐operation between nurses with different responsibilities	3 (1.6)	36 (19.8)	3 (1.6)	—	9 (16.1)	1 (1.8)	2 (3.4)	13 (22.0)	—	1 (1.5)	14 (20.9)	2 (3.0)
Care management and work environment (Poor/Acceptable/Good^a^), *N*/*n* (%)												
Capability to manage and lead nursing care	13 (7.1)	157 (86.3)	9 (4.9)	2 (3.6)	48 (85.7)	5 (8.9)	5 (8.5)	50 (84.7)	—	6 (9.0)	59 (88.1)	2 (3.0)
Work environment	17 (9.3)	162 (89.0)	3 (1.6)	7 (12.5)	46 (82.1)	3 (5.4)	4 (6.8)	55 (93.2)	—	6 (9.0)	61 (91.0)	—

Abbreviations: *n* = a sample of the total number; *N* = total number.

^a^ Variables recoded to: Poor = Very poor/poor; Acceptable = Acceptable; Good = Good/Very good.

^b^ Variables recoded to: Never = Never/Rarely; Sometimes = Occasionally/A moderate amount; A great deal = A great deal.

### 3.7 Organisational Modulators

A total of 623 organisational contextual cues were identified during the 182 observations of RNs alone. Signs or verbal remarks of stress and tiredness were noted in 27.5% and 12.6% of observations, respectively. RNs' ability to lead and manage care was the most frequently observed cue and mainly rated as ‘acceptable’ in 86.3% of observations but ‘poor’ in 7.1%. Work environments were marked as ‘poor’ in 9.3% (Table 7). Field notes described visible fatigue and reduced attentiveness during care.

The RN shows signs of stress and remarks this to the observer. The RN does not take off their jacket when entering the home, wearing this for the entire visit (ca. 10 min). The RN also asks the older person very briefly if they want any food; when the answer is “no” the RN does not follow up on the answer; nothing more is said. The RN is tasked to make psychosocial observations during the visit—asks briefly about the older person's day/mood, but does not follow up on the answer; “uhm” is the only reply. Then somewhat abruptly the RN says, “Alright, now we must go. Have a good night”. Still wearing the jacket, the RN leaves. (Field note #67)

The RN shows clear signs of tiredness, yawning and sighing several times during the observation/visit. The RN sits quietly during most of the dialysis treatment, approximately one hour, while the TV is on. The RN does ask “How is it going?” but does not follow up on the answer from the older person. The RN shows no sign of registering that the bandage around the dialysis entry point needs changing (Field note #219)

Transformational leadership cues were identified 179 times, primarily ‘goal‐oriented’, ‘democratic’ and ‘intellectual stimulation’, consistent across all teams. Transactional leadership cues (*n* = 177) included ‘identifies with shared team values’ and ‘makes decisions quickly’. Variations between teams emerged: Team B emphasised managerial focus, and Team C showed more cues related to leading change (Table 7).

**TABLE 7:** Leadership styles as contextual modulators of practice among registered nurses.

Leadership style	Teams A–C	Team A	Team B	Team C
	*N* = 182	*n* = 56	*n* = 59	*n =* 67
Transformational leadership cues, *N*/*n* (%)				
Catalyst for change	31 (17.0)	9 (16.1)	13 (22.0)	9 (13.4)
Democratic approach (shares responsibilities)	45 (24.7)	13 (23.2)	16 (27.1)	16 (23.9)
Goal‐orientated (sets clear expectations)	47 (25.8)	14 (25.0)	16 (27.1)	17 (25.4)
Intellectual stimulator (encourages new ideas)	31 (17.0)	8 (14.3)	12 (20.3)	11 (16.4)
Inspirational	14 (7.7)	4 (7.1)	5 (8.5)	5 (7.5)
Visionary (articulates a vision)	11 (6.0)	6 (10.7)	3 (5.1)	2 (3.0)
Transactional leadership cues, *N*/*n* (%)				
Identifies with shared team values	46 (25.3)	14 (25.0)	16 (27.1)	16 (23.9)
Focus on managerial tasks	30 (16.5)	14 (25.0)	9 (15.3)	7 (10.4)
Goal‐oriented (rewards completed tasks)	14 (7.7)	5 (8.9)	7 (11.9)	2 (3.0)
Leads change	33 (18.1)	10 (17.9)	13 (22.0)	10 (14.9)
Makes decision quickly (effective in crisis)	42 (23.1)	12 (21.4)	16 (27.1)	14 (20.9)
Motivational (provides rewards for task completion)	12 (6.6)	5 (8.9)	6 (10.2)	1 (1.5)

Abbreviations: *n* = a sample of the total number; *N* = total number.

All teams were observed to operate within a hierarchical organisational model, yet the nursing teams exhibited flatter internal structures supporting autonomy, collaboration, and open communication. The prevailing subculture was constructive—encouraging goal pursuit, innovation, and mutual support—though traces of passive or defensive tendencies remained, including conformity and conflict avoidance. Field notes also indicated emerging shifts toward a more open and reflective culture

The RN talks with a colleague about increasing the medication for a patient who is experiencing difficulty breathing and follows up on the plan regarding the dosage adjustment and administration form. The RN demonstrates a democratic approach and goal‐oriented attitude as she discusses with her colleague how to proceed with the medication plan. She ensures that they have clarified the goals and expectations of the plan, emphasising the importance of following up and supporting other colleagues in its implementation. (Field note #14)

## 4 Discussion

This study aimed to describe nurses' SoP in home‐based care, focusing on enacted care activities, clinical decision‐making, professional autonomy, and contextual modulators of practice. To understand and frame the findings from our observations within home‐based care, we used the conceptual framework developed for this study (Figure 1). The framework comprised the following core constructs: nurses' roles, functions, and clinical decision‐making, their enactment in practice, and the contextual modulators shaping practice. Previous research has highlighted the complexity and competence demands of home‐based nursing care (Rusli et al. 2023). However, such work primarily synthesises expected competencies rather than examining how SoP is enacted in situ. The present study extends this by showing how contextual modulators are associated with observable nursing practice in real time.

Contextual modulators of practice emerged as central to understanding how nurses' SoP was enacted in home‐based care. Across observations, nurses frequently encountered interruptions, time pressure, stress, and varying working conditions, which influenced how care activities, clinical decision‐making, and professional autonomy were realised in situ. These findings are supported by previous research (Brenne et al. 2022; Sandberg et al. 2026). They indicate that SoP cannot be understood solely as an individual or role‐based phenomenon. Rather, it appears to be a system‐level process shaped by the dynamic interplay between contextual modulators of practice on an individual and organisational level (see Table S1). Although this study did not aim to establish causal relationships between modulators and care outcomes, the observed patterns align with previous research demonstrating that such modulators can influence both care processes and patient outcomes (Li et al. 2024). Taken together, the findings underscore the importance of addressing context, both in focus and through appropriate methods, when seeking to understand or strengthen practice in home‐based care settings.

Within this practice, the observed pattern of care activities reflects how nurses prioritise and enact care. As anticipated, our findings illustrate that independent work during brief home visits involving multiple care activities, e.g., physical care and medication management, requires nurses' advanced clinical judgement in complex, time‐sensitive situations. Consistent with prior research (Fjørtoft et al. 2021; Martinsen et al. 2018), nurses in our study devoted most of their limited time to medication administration and fundamental physical care, with fewer psychosocial care activities observed. Nevertheless, field notes indicated nurses' efforts to sustain respectful, person‐centred interactions and use the nurse–patient relationship despite time pressures. These findings contrast with two recent observational studies (Nordaunet et al. 2025; Sandberg et al. 2026), using the Fundamentals of Care framework, emphasising relational and psychosocial aspects, which reports greater psychosocial focus in enacted nursing practice, raising the question of whether differences reflect practice or the observational framework. Notwithstanding, our findings can be interpreted to align with evidence across contexts suggesting that nursing practice may be constrained by contextual modulators and potentially increasing the risk for missed or rationed care (Gong et al. 2025). Despite longstanding recognition of these challenges and their negative consequences (Kalánková et al. 2020), there is limited evidence of meaningful organisational reform. While additional resources are needed, alternative strategies are also required. Therefore, practical, evidence‐based approaches to support nurses in managing these contextual modulators are essential.

While the nursing process remains a widely used framework for structuring and operationalising clinical decision‐making, its applicability and linearity have been questioned, particularly in complex community‐ and home‐based care settings. Nevertheless, clinical decision‐making aligned with the nursing process remains a central component of RN practice, particularly in caring for populations with fluctuating and complex needs. Our findings indicate that observable decision‐making focused mainly on assessment of care needs and the clinical situation, with evaluation less observationally evident—consistent with prior studies across settings (Nordaunet et al. 2025; Sandberg et al. 2026). These findings should be considered in relation to contextual modulators of practice, which may compel nurses to prioritise immediate or salient challenges, potentially restricting the comprehensive enactment of clinical decision‐making within system‐level constraints. Regardless of cause, supporting RNs to enact all phases of the decision‐making process, particularly given their autonomous responsibility to analyse and evaluate care, both their own and that provided by non‐RNs (RCN 2023; Richards and Borglin 2019) seems important. Neglecting this part of RNs' role may lead to adverse outcomes for older people, including increased frailty, reduced functional ability, higher fall risk, and greater likelihood of hospitalisation or institutionalisation.

Although Lydia Hall was among the first to conceptualise nursing as a process and to clarify the RN's autonomous role in the 1950s (as cited in Yura and Walsh 1973), our findings indicate that nurses continue to engage predominantly in interdependent care activities, that is, activities informed or decided in collaboration with other professionals (Wilkinson 2011). Notable, Brenne et al. (2022) found that RNs describe their work as requiring a high degree of independence and autonomous decision‐making. In Norwegian home‐based care, however, predefined lists of care activities often structure visits and may constrain autonomy. One might therefore argue that the autonomy observed in this study is substantial within these constraints. Nonetheless, our findings underscore continued importance of supporting RNs' autonomous role in home‐based care, in line with Hall's original vision. While others (e.g., RCN 2023) have questioned the extent to which any healthcare professional can act autonomously, our results suggest that RNs do exercise professional autonomy within their SoP, although the extent to which autonomy was enacted appeared influenced by contextual modulators of practice.

The inherently challenging nature of home‐based care limits RNs' opportunities to exercise leadership. When observed, leadership was primarily characterised as transformational—setting clear expectations, sharing responsibilities, and encouraging initiative—with some less consistent transactional elements, such as rapid decision‐making. Although observations of RN leadership were limited, the findings highlight ongoing challenges RNs face in enacting this responsibility in home‐based care, potentially requiring other leadership skills compared to different contexts. Alongside time constraints, potentials for care rationing, and the partial use of the nursing process, these results underscore the need for RNs to be supported as competent, confident, and empowered leaders in complex practice environments.

### 4.1 Methodological Considerations

A multi‐method observational design was employed, incorporating structured direct observations (Fix et al. 2022) and textual data to balance structure with the flexibility needed to describe the complex phenomenon of SoP in home‐based care. Combining numerical and textual data enhanced both analytical depth and breadth, contributing to the study's overall credibility (Patton 2015). A key aim was to expand contextual understanding by examining modulators of nurses' SoP, with a view to identifying uncertainties and practical considerations essential for developing future interventions with real‐world relevance (cf. Skivington et al. 2021).

The recruitment strategy was designed to enhance methodological rigour in this complex setting. Measures included clear participant information, familiarisation days prior to observations, and a top‐down approach to secure managerial support and formalise recruitment. A sequential multi‐stage recruitment process balanced representativeness with the inclusion of information‐rich participants. The involvement of a large home healthcare service allowed sampling across multiple teams, thereby capturing a mix of RNs and non‐RNs. Random team selection helped reduce bias, while purposive sampling (Patton 2015) of nurses and care recipients supported alignment with the study aim. The inclusion of both RNs and non‐RNs enabled a more comprehensive understanding of nurses' SoP as it is enacted within the broader care context.

Data were collected by a single non‐participant observer, with careful attention to minimising the Hawthorne effect (Franke and Kaul 1978). Participants were informed about the study, but not the specific focus of each observation, to reduce behaviour change. Regular breaks were scheduled to reduce participant burden and preserve data quality. Observations spanned mornings and afternoons throughout the week, capturing a wide range of care activities. Although the use of a single observer might be a limitation, observer bias was mitigated through a structured, piloted protocol grounded in established theoretical frameworks and aligned with validated observational tools (Nordaunet et al. 2025; Sandberg et al. 2026). While observations were conducted across three teams within a large municipal home healthcare service, organisational arrangements, staffing patterns, and role configurations may vary across municipalities and healthcare systems. Consequently, the findings should be interpreted in relation to the Norwegian home‐based care context. At the same time, the data provide a Nordic cross‐sectional snapshot of nursing practice, and transferability to other settings should therefore be considered in light of contextual differences.

## 5 Conclusions

This study advances understanding of nurses' SoP, its contextual modulators, and the implications for their roles (responsibilities) and functions (activities, tasks, interventions) within home‐based care. Our findings indicate that nurses frequently undertook care activities extending beyond the predefined plan for home visits. RNs particularly focused on medication management, whereas non‐RNs concentrated on aspects related to activities of daily living. A critical gap was identified in the enactment of the nursing process. RNs primarily emphasised the assessment phase, non‐RNs focused on implementation, and both groups largely omitted the evaluation phase. This omission may compromise the integrity of the nursing process and increase the risk of adverse outcomes for older people receiving home‐based care. These findings should be interpreted in light of contextual modulators, such as stress, interruptions, and suboptimal working conditions, which clearly influenced the nurses' ability to enact their full SoP. Overall, the results highlight the need to strengthen organisational and professional support systems to enable nurses, particularly RNs, to exercise their full SoP and deliver safe, comprehensive, and person‐centred care in home‐based settings.

### 5.1 Implications for Practice and Research

The findings underscore the importance of enhancing RNs' autonomous decision‐making and strengthening their capacity to apply the nursing process across all phases. Supporting RNs in clinical leadership roles may help ensure consistent assessment, implementation, and evaluation of care, while organisational interventions to reduce stress, manage interruptions, and improve working conditions are essential to sustaining high‐quality nursing practice. Efforts to operationalise and embed theoretical innovations should be guided by empirical evidence on how the nursing process functions in real‐world care environments. Addressing these challenges is crucial to align home‐based nursing practice with professional standards and optimise patient safety and well‐being.

Our study reinforces the view that nurses' SoP should be understood as a system‐level process rather than isolated individual behaviour. There remains a need for in situ methodologies, particularly direct observation, to capture the nuanced, context‐dependent nature of nurses' practice, including aspects often inaccessible through self‐report approaches such as interviews or questionnaires. Future research should investigate broader contextual modulators shaping nurses' SoP, including organisational culture, team dynamics, and nursing subcultures to support the development of more contextually grounded, practice‐informed frameworks for policy, education, and workforce development.

## Author Contributions

All authors contributed to the study conception and design and approved the final manuscript. **Ida Røed Flyum:** conceptualisation, methodology, formal analysis, data curation, investigation and writing – original draft. **Gunilla Borglin:** supervision, conceptualisation, methodology, formal analysis, writing – original drafting, reviewing, and editing. **Anna Josse Eklund:** supervision, methodology, formal analysis, writing – review and editing. **Veronica Pavedahl:** supervision, writing – review and editing. **Edith Roth Gjevjon:** supervision, conceptualisation, methodology, formal analysis, investigation, writing – review and editing.

## Funding

The authors have nothing to report.

## Disclosure

This research report is part of the PhD project DECIDE (Developing Expertise in Care and Informed Decision‐making), which is included in the Nordic collaboration and the programmatic research platform Continuity for Quality of Care in Nursing (CARE) at Lovisenberg Diaconal University College, Norway, and Karlstad University, Sweden. The DECIDE project employs a multi‐method design focusing on nurses' scope of practice (SoP), including their decision‐making processes and the contextual modulators that influence them – primarily in the context of home‐based care. The project uses functional ability and frailty among older people as clinical examples of conditions that contribute to complex care needs. DECIDE is being conducted between 2021 and 2026.

Prior to submission, a GDPR‐compliant version of ChatGPT was used to assist with readability, language refinement, and sentence structure. The tool was used solely during the writing process to optimise the final draft, including after revisions of the manuscript. On both occasions, the authors critically reviewed and edited all content to ensure that the AI's contributions were limited to minor language improvements and did not affect the substance of the work. The authors take full responsibility for the content of the published article.

## Ethics Statement

This study adhered to relevant ethical guidelines (World Medical Association 2024) and was conducted in accordance with the European Union's General Data Protection Regulation. Ethical approval was obtained from the Regional Committees for Medical and Health Research Ethics and the Norwegian Agency for Shared Services in Education and Research (IDs 733476 and 349256), as well as from the Research Ethics Committee at Karlstad University and the Swedish Ethical Review Authority (IDs C2024/636 and 2025‐00816‐01). The study was part of the DECIDE project, which includes patient and public involvement at programme level.

## Conflicts of Interest

The authors declare no conflicts of interest.

## Supporting information


**Table S1:** Conceptual framework and operationalisation this study's core constructs.

**Table S2:** Mixed Methods Appraisal Tool—MMAT.

**Table S3:** Observational protocol and data collection structure.

## Data Availability

The raw data that supports the findings of this study is not available due to confidentiality and ethical restrictions including data would not be shared. The developed and tested observational protocol is available upon reasonable request from the corresponding author.
