# Supplementary material for: Nurses' Scope of Practice in Home‐Based Care: An Observational Study of Care Activities, Clinical Decision‐Making, and Contextual Modulators—The DECIDE Project
Source: J Clin Nurs. 2026 Jul 22;35(12):5386–99. doi: 10.1111/jocn.70451 (PMC13744246; doi:10.1111/jocn.70451)
Supplement: Supplementary file 1 [file JOCN-35-5386-s001.pdf]

Supplementary Table S1. Conceptual framework and operationalisation for this study's core constructs

Components	Operationalised as	Data sources	Observational cues / triggers	Levels	Basis
Core context					
Nurses	Registered nurses (RNs) and non-registered nurses (non-RNs)	Observation protocol	Identification of nurse delivering care	Individual	International Council of Nurses (2025)
Home-based care	Nursing care delivered in patients' homes	Contextual documentation	Care activity occurring in home setting	Contextual	Higgs et al. (2008); Royal College of Nursing (2023)
Complex care needs	≥2 chronic conditions (multimorbidity)	Inclusion criteria	RN-confirmed eligibility	Contextual (patient)	Navickas et al. (2016)
Scope of Practice					
Scope of Practice (SoP)	Role (responsibility), function (activities), and clinical decision-making	Observation protocol + field notes	Engagement in care activities and/or decision-making	Individual	International Council of Nurses (2025); Malone & Cliffe (2018); Royal College of Nursing (2023)
Practice enactment					
Care activities (VIPS)	Physical, psychosocial, and composite/medical needs	Observation protocol	Activity initiated corresponding to VIPS keyword	Individual (activity)	Ehnfors et al. (1992; 2015)
Clinical decision-making (nursing process)	Assessment, analysis/diagnosis, planning, implementation, evaluation	Observation protocol + field notes	Observable action reflecting a phase of the process	Individual (process)	Wilkinson (2011)
Performance level of care	Compensatory, partially compensatory, collaborative care	Observation protocol	Degree of patient involvement in care	Individual (activity)	Henderson (1997)
Professional autonomy	Independent, interdependent, or dependent decision-making	Observation protocol	Who initiates and decides care activity	Individual	Wilkinson (2011)
Contextual modulators of practice					
Individual modulators (dynamic)	Capability to perform, assess, concentrate, and decide	Observation protocol + field notes	Observable cues (e.g., interruptions, hesitation)	Individual	Lister et al. (2020)
Organisational structure	Organisation of work, roles, responsibilities, and coordination within home-based care	Field notes (aggregated)	Inferred from patterns in task allocation, coordination, and workflow across observations	Contextual (organisational)	Orvik (2022)
Organisational factors (dynamic)	Stress, fatigue, communication, cooperation, work environment	Observation protocol + field notes	Observable organisational influences during care	Individual/contextual	Lister et al. (2020)
Leadership style	Transformational and transactional leadership behaviours	Observation protocol + field notes	Observable leadership-related actions (e.g., coordination, delegation)	Individual (RN)	Cope & Murray (2017)
Nursing unit culture	Patterns of shared behaviours, communication, and collaboration across the team	Field notes (aggregated)	Inferred from repeated patterns across observations (not a single events)	Team (derived)	Callen et al. (2009); Mannion & Davies (2018)
