# Supplementary material for: Nurses' Scope of Practice in Home‐Based Care: An Observational Study of Care Activities, Clinical Decision‐Making, and Contextual Modulators—The DECIDE Project
Source: J Clin Nurs. 2026 Jul 22;35(12):5386–99. doi: 10.1111/jocn.70451 (PMC13744246; doi:10.1111/jocn.70451)
Supplement: Supplementary file 2 [file JOCN-35-5386-s002.pdf]

Supplementary Table S3. Observational protocol and data collection structure

Observational protocol section	*Operationalisation and description*
Section A-B<br>*(Units of observation)*	*Care activities* corresponding to the VIPS model (Well-being, Integrity, Prevention, and Safety).<br>Care activities related to *physical needs (10 cues/items):*<br>*Communication* – Ability to communicate and express needs, impairment in functions (sight, hearing, speech).<br>*Knowledge/development* – Cognitive status. Need for information or education. Understanding of health/disease.<br>*Breathing/circulation* – Respiratory distress, cough, bleeding, oedema, cyanosis, temp, etc. Self-care/aids; mattress, support stockings, etc.<br>*Nutrition* – Appetite, thirst, diet, eating habits, oral health, under- or overweight, fluid- and electrolyte balance, blood sugar, gastrointestinal issues. Nausea, vomiting, dehydration or overhydration. Self-care/aids; special diet or dentures.<br>*Elimination* – Elimination of urine or stool, bladder or bowel incontinence, constipation, diarrhoea, stoma. Self-care/aids; diet, laxatives, etc<br>*Skin/tissues/wounds* – Changes in skin, mucous membranes, hair, nails, colour, cleanliness, appearance, function, and changes. Pressure ulcers or lacerations; localisation, colour, stage of healing. Self-care/aids; medications, mattress, etc.<br>*Activity (ADL/mobility)* – Personal hygiene, getting dressed, housework, etc. Physical, social, intellectual (in)activity. Self-care/aids; mobility aids.<br>*Sleep* – Sleep and rest. Intermittent or chronic tiredness, exhaustion, etc. Facilitating factors. Self-care/aids; medications, habits, etc.<br>*Pain/perception* – Acute/chronic pain or discomfort. Both subjective and objective data; Localisation, character, intensity, and the older persons' words and expressions of pain. Behaviour related to pain; tears, not able to stay still, protective movements. Triggering or relieving factors. Self-care/aids; warming pack, massage, etc.<br>*Sexuality/reproduction* – Side-effects of medications.<br>Care activities related to *psychosocial needs (3 cues/items):*<br>*Emotions* – Experiences, feelings, reactions; apathy, sorrow, aggression, loneliness, happiness, motivation, endurance, experience of meaning and coherence, stress, stress tolerance, coping. Expectations and reflections.<br>*Relations* – Roles and interactions, perception of self, personal integrity, social support and network, social capacity, family difficulties, loneliness and responsibility. Alcohol, substance abuse.<br>*Spiritual/cultural wellbeing* – Culture, religion. Existential question, death, suffering, over-all wellbeing. Balance of health, ill health, resources and needs.<br>Care activities related to *composite or medical needs (3 cues/items):*<br>*Composite assessment* – Assessment using scales or instruments such as comprehensive geriatric assessment (CGA), to assess the care recipient's combined status across multiple keywords.<br>*Medications* – Administration, problems, side-effects, interactions, other needs, etc. Need for other prescriptions.<br>*Medical assessment* – Medical assessment or diagnosis by the RN, according to competence and authorisation.
Section C-D	*Clinical decision-making* (nursing process), that is: Assessment, Analysis and diagnosis, Planning and setting goals, Implementation, and Evaluation.
Section E-F	The *nursing activities' performance level*, that is, the compensatory level: Compensating care, partially compensating care, or collaborative care.<br>The *nurses' level of professional autonomy* – in the activity, that is, dependent, interdependent, or independent.
Section G-I	Individual and organisational *modulators of context* – such as practical skill, awareness, stress, work environment, and leadership style (transformational and transactional).
Additional data gathered	Information was also gathered related to organisational structures and cultural influences per team.
