# Supplementary material for: Nurses' Scope of Practice in Home‐Based Care: An Observational Study of Care Activities, Clinical Decision‐Making, and Contextual Modulators—The DECIDE Project
Source: J Clin Nurs. 2026 Jul 22;35(12):5386–99. doi: 10.1111/jocn.70451 (PMC13744246; doi:10.1111/jocn.70451)
Supplement: Supplementary file 3 [file JOCN-35-5386-s003.pdf]

Supplementary Table S2.

Mixed Methods Appraisal Tool (MMAT)

Category of study designs	Methodological quality criteria	Responses			
		Yes	No	Can't tell	Comments
Screening questions (for all types)	S1. Are there clear research questions?	√			Page 4
	S2. Do the collected data allow to address the research questions?	√			Page 6
	Further appraisal may not be feasible or appropriate when the answer is 'No' or 'Can't tell' to one or both screening questions.				
1. Qualitative	1.1. Is the qualitative approach appropriate to answer the research question?	√			Page 4
	1.2. Are the qualitative data collection methods adequate to address the research question?	√			Page 6
	1.3. Are the findings adequately derived from the data?	√			Page 7-11
	1.4. Is the interpretation of results sufficiently substantiated by data?	-	-		No interpretation used
	1.5. Is there coherence between qualitative data sources, collection, analysis and interpretation?	√			Page 4-11, quotes
2. Quantitative randomized controlled trials	2.1. Is randomization appropriately performed?				*N/A*
	2.2. Are the groups comparable at baseline?				*N/A*
	2.3. Are there complete outcome data?				*N/A*
	2.4. Are outcome assessors blinded to the intervention provided?				*N/A*
	2.5 Did the participants adhere to the assigned intervention?				*N/A*
3. Quantitative non-randomized	3.1. Are the participants representative of the target population?				*N/A*
	3.2. Are measurements appropriate regarding both the outcome and intervention (or exposure)?				*N/A*
	3.3. Are there complete outcome data?				*N/A*
	3.4. Are the confounders accounted for in the design and analysis?				*N/A*
	3.5. During the study period, is the intervention administered (or exposure occurred) as intended?				*N/A*
4. Quantitative descriptive	4.1. Is the sampling strategy relevant to address the research question?	√			Page 5
	4.2. Is the sample representative of the target population?	√			Page 5
	4.3. Are the measurements appropriate?	√			Page 6-7
	4.4. Is the risk of nonresponse bias low?	-	-		*N/A*
	4.5. Is the statistical analysis appropriate to answer the research question?	√			Page 7
5. Mixed methods	5.1. Is there an adequate rationale for using a mixed methods design to address the research question?				*N/A*
	5.2. Are the different components of the study effectively integrated to answer the research question?				*N/A*
	5.3. Are the outputs of the integration of qualitative and quantitative components adequately interpreted?				*N/A*
	5.4. Are divergences and inconsistencies between quantitative and qualitative results adequately addressed?				*N/A*
	5.5. Do the different components of the study adhere to the quality criteria of each tradition of the methods involved?				*N/A*

Hong QN, Pluye P, Fàbregues S, Bartlett G, Boardman F, Cargo M, Dagenais P, Gagnon M-P, Griffiths F, Nicolau B, O'Cathain A, Rousseau M-C, Vedel I. Mixed Methods Appraisal Tool (MMAT), version 2018. Registration of Copyright (#1148552), Canadian Intellectual Property Office, Industry Canada.
